# Supplementary material for: Comparison of efficacy and safety of single versus repeated intra-articular injection of allogeneic neonatal mesenchymal stem cells for treatment of osteoarthritis of the metacarpophalangeal/metatarsophalangeal joint in horses: A clinical pilot study
Source: PLoS One. 2019 Aug 29;14(8):e0221317. doi: 10.1371/journal.pone.0221317 (PMC6715221; doi:10.1371/journal.pone.0221317)
Supplement: S1 Table — (DOCX) [file pone.0221317.s001.docx]

| ID  horses | Sex | Age | Race | Activity | Limb | Days between diagnosis and treatment | Arthroscopy bf inj. | Treatments before injection | Clinical score inclusion | Treatment group |
| --- | --- | --- | --- | --- | --- | --- | --- | --- | --- | --- |
| 1 | F | 10 | KWPN | Jumping | RF | 114 |  | Rest , oral NSAID | 14 | MSC1 |
| 2 | S | 4 | Selle Francais | Jumping | RF | 538 |  | Rest | 8 | MSC2 |
| 3 | F | 6 | SBS | Jumping | LF | 455 |  | Rest, oral chondroprotectors | 8 | MSC1 |
| 4 | G | 5 | BWP | Jumping | RF | 174 |  | Rest | 14 | MSC1 |
| 5 | F | 15 | Selle Francais | Jumping | LH | 234 |  | IA injection with Triamcinolone | 16 | MSC1 |
| 6 | S | 2 | StandardBred | Trotting | LH | NK | yes |  | 11 | MSC2 |
| 7 | G | 11 | Selle Francais | Jumping | LF | NK | yes |  | 4 | MSC2 |
| 8 | F | 9 | Selle Francais | Jumping | LF | NK | yes |  | 7 | MSC1 |
| 9 | G | 6 | Mixed Breed | Jumping | LF | 890 |  | IA injection with Triamcinolone and Hyaluronic acid | 8 | MSC2 |
| 10 | G | 9 | Selle Francais | Jumping | LF | 563 |  | IA injections with Triamcinolone and IRAP | 11,5 | MSC2 |
| 11 | F | 9 | Suiss Warmblood | Jumping | RH | NK | yes (3 months before) |  | 4 | MSC1 |
| 12 | G | 13 | BWP | Jumping | RF | NK |  | IA injection with Hyaluronic acid | 10 | MSC2 |
| 13 | G | 8 | Paint Horse | Western | RF | 517 |  | Hoof trimming, tiludronate | 5 | MSC1 |
| 14 | F | 7 | Selle Français | Jumping | LF | NK | yes | Rest | 8 | MSC1 |
| 15 | G | 12 | Oldenbourg | Dressage | LH | NK | yes | IA injection with PRP | 6 | MSC1 |
| 16 | G | 14 | Arabian | Endurance Riding | RF | 191 |  |  | 8 | MSC2 |
| 17 | G | 17 | Lusitano | Pleasure | RF | NK |  |  | 2 | MSC2 |
| 18 | G | 5 | Standard Bread | Trotting | LF | NK | yes | IA injection with Triamcinolone and Hyaluronic acid | 11 | MSC2 |
| 19 | F | 10 | Anglo Arabian | Jumping | RF | 1080 |  | Tildren, Rest | 11 | MSC1 |
| 20 | F | 10 | Selle Francais | Jumping | LF | NK |  | Rest, IA injection with steroids twice | 6 | MSC2 |
| 21 | G | 7 | Selle Francais | Jumping | LH | NK | yes |  | 6 | MSC2 |
| 22 | G | 7 | Selle Francais | Jumping | LF | 168 | yes |  | 9 | MSC2 |
|  |  |  |  |  |  |  |  |  |  |  |
| 23 | F | 3 | Thouroughbred | Jumping | LF | NK |  |  | 6 | MSC2 |
| 24 | G | 7 | Friesian | Pleasure | LF | 523 |  | Tildren | 12 | MSC 1 |
| 25 | G | 6 | Thoroughbred | Racing | LF | 168 |  | Rest | 10 | MSC 1 |
| 26 | F | 14 | Selle Francais | Jumping | RF | 487 |  | IA injections with IRAP and Hyaluronic acid | 9 | MSC1 |
| 27 | F | 7 | Czech Warmblood | Jumping | LH | foal |  | Rest | 7 | MSC2 |
| 28 | F | 15 | Selle Francais | Jumping | RF | 252 |  | Rest, corrective shoeing | 8 | MSC1 |
